# Immune alterations in vestibular neuritis: a retrospective database pilot study on T and B lymphocyte profiles and cytokine levels

**DOI:** 10.3389/fneur.2026.1749781

**Published:** 2026-01-16

**Authors:** Zhaohui Song, Yuchuan Ding, Wesley Kohls, Jing Feng, Huimin Fan, Pan Gu, Xiaokun Geng

**Affiliations:** 1 Department of Neurology, Beijing Luhe Hospital, Capital Medical University, Beijing, China; 2 Department of Neurosurgery, Wayne State University School of Medicine, Detroit, MI, United States; 3 China-America Institute of Neuroscience, Beijing Luhe Hospital, Capital Medical University, Beijing, China

**Keywords:** DHI score, IL-17A, IL-6, immune, NK cells, vestibular neuritis

## Abstract

**Background:**

Vestibular neuritis (VN) etiology remains elusive, with hypotheses suggesting viral infection, non-infectious inflammation, or immune responses. Evaluating T and B Lymphocyte Subsets and cytokines gives a comprehensive snapshot of the body’s immune function and inflammatory state. But comprehensive studies focusing on the T and B lymphocyte subsets and cytokine levels in VN are limited. This study aims to assess the T and B lymphocyte subsets and cytokine expressions in the blood of VN patients. This study shed light on its pathogenesis and provided valuable hematological markers for clinical prognosis.

**Methods:**

A retrospective analysis was conducted on subjects diagnosed with VN. Patients included exhibited acute, first-episode, persistent vertigo with accompanying symptoms of nausea, vomiting, postural instability, specific nystagmus, and positive head impulse test results. Exclusion criteria included hearing impairments, prior vestibular disorders, recent steroid therapy, and autoimmune conditions. Patients underwent blood tests (T and B lymphocyte subsets, cytokines) and DHI evaluations within days of onset, with a secondary assessment at discharge. Healthy individuals served as controls.

**Results:**

The study included 25 individuals with VN (aged 34–73 years, 13males) and 25 healthy controls (aged 33–74 years, 7males). VN patients exhibited elevated levels of total B lymphocytes, helper/inducer (CD3^+^CD4^+^) T cells, and the helper/suppressor (CD4^+^/CD8^+^) T cell ratio all of which were statistically significant compared to the control group. In contrast, reduced levels of total T lymphocytes, suppressor/cytotoxic T cells, and natural killer cells were observed. Elevated Interleukin-6 levels and decreased Interleukin-17A levels were seen in the VN group. There are differences in the levels of CD3^+^CD4^+^ T cells and CD4^+^/CD8^+^ T cell ratio among patients in the three groups of mild, moderate, and severe, with the severe group significantly higher than the mild group. Admission levels of CD3^+^CD4^+^ T cells showed positive correlations with the DHI-Functional score within 1–3 days. Admission levels of CD3^+^CD4^+^ T cells and CD4^+^/CD8^+^ T cell ratio are positively correlated with all DHI scores at discharge.

**Conclusion:**

Immunological perturbations are implicated in pathogenesis of VN. Evaluation of these immune markers could offer insights into prognostic outcomes for VN patients, leading to development of therapeutic strategies.

## Introduction

Vestibular neuritis (VN) is an acute vestibular syndrome resulting from damage to one side of the vestibular nerve (1, 2). VN manifests with severe symptoms typically lasting 3–5 days, with over 30% of affected individuals suffering dizziness for more than a year (1–3). While its etiology remains elusive, potential culprits range from viral infections and non-infectious inflammation to immune-mediated processes (2–8). With growing focus on the latter two, especially the role of the immune response in VN’s pathogenesis, there is a pressing need to investigate further (5–10).

T and B lymphocytes play a pivotal role in the acquired immune response, as they uniquely recognize and react to specific antigenic epitopes. Evaluating their subsets gives a comprehensive snapshot of the body’s immune function, thereby evaluating immune status and disease processes. Among these subsets, the CD3^+^CD4^+^ T cells, CD3^+^CD8^+^ T cells, CD4^+^/CD8^+^ ratio, and NK cell count offer invaluable insights into the immune system’s activity, equilibrium, and functional status (11, 12). Moreover, cytokines, the proteins secreted by immune cells, act as cellular communicators, modulating immune activities and inflammation (13). The measure of specific cytokines like tumor necrosis factor (TNF), interleukins (IL), interferons (IFN), and chemokines can shed light on the body’s immune and inflammatory state (14–17). Notably, despite their potential significance, comprehensive studies focusing on the T and B lymphocyte subsets and cytokine levels in VN are limited (8, 10). The association between these immune markers and VN’s severity and prognosis remains to be determined.

Therefore, this study aimed to comprehensively profile the alterations in peripheral blood T and B lymphocyte subsets and cytokine levels in patients with VN. Furthermore, we sought to explore the associations between these immune markers and clinical disease severity as well as short-term functional recovery, assessed by the Dizziness Handicap Inventory (DHI). We hypothesize that specific immune factors may serve as potential biomarkers for evaluating disease severity and predicting clinical outcomes, which could inform future therapeutic strategies for VN.

## Methods

### Study design

We retrospectively analyzed patients admitted to the Department of Neurology at Beijing Luhe Hospital, affiliated with Capital Medical University, between January 2022 and June 2023. We collected and analyzed this data from August 2023 to December 2023. The studies were reviewed and approved by Ethics Committee of Beijing Luhe Hospital. We followed the Strengthening the Reporting of Observational Studies in Epidemiology (STROBE) guidelines to enhance the transparency and completeness of our study report. The study design, data collection methods, and analysis procedures were structured according to the STROBE criteria to ensure comprehensive and accurate reporting of our observational study (18).

### Inclusion and exclusion criteria

These patients were initially identified based on the clinical criteria for VN and were within 1–2 days of symptom onset. Patients were included in the study if they met the following criteria: (1) ages range 18–80 years old; (2) acute, first-episode, persistent vertigo, accompanied by nausea, vomiting, and postural instability; (3) presence of horizontal-torsional spontaneous nystagmus directed toward the unaffected side; (4) positive head impulse test toward the opposite side; and (5) absence of hearing loss and other focal neurological symptoms or signs. For comparison, healthy individuals were also recruited for the study. Patients and health subjects were excluded if they met any of the following criteria: (1) presence of hearing impairment, tinnitus, or other focal neurological symptoms or signs, to ensure inclusion of a typical vestibular neuritis phenotype; (2) a history of vestibular disorders, including vestibular neuritis, Ménière’s disease, or vestibular migraine; (3) steroid or hormone therapy within the preceding month, to avoid potential pharmacological immunomodulation affecting lymphocyte subsets and cytokine measurements; (4) coexisting autoimmune diseases, such as rheumatoid arthritis, systemic lupus erythematosus, or multiple sclerosis, to isolate vestibular neuritis–specific immune alterations. By applying these criteria, we aimed to gather a comprehensive cohort of acute VN patients for a robust analysis. Healthy subjects were excluded if they had a history of acute infections in the past 2 weeks (including upper respiratory or gastrointestinal infections, etc.), which could induce transient systemic inflammatory responses and confound immune parameters and were not receiving any ongoing treatments. These exclusion criteria were applied to minimize potential contradictory factors and to ensure accurate assessment of immune alterations associated with vestibular neuritis. All patients in our study underwent comprehensive vestibular function testing upon admission, which included the caloric test, video Head Impulse Test (vHIT), Vestibular-Evoked Myogenic Potentials (VEMP), and Subjective Visual Vertical (SVV). These tests confirmed unilateral vestibular dysfunction in all patients.

### Outcome measures

#### Assessment of T and B lymphocyte subsets

A 3 mL-fasting blood sample was obtained from the antecubital vein of a study participants at 8 a.m. This peripheral blood was combined with 2 mL of Ethylenediaminetetraacetic Acid (EDTA) as an anticoagulant. T lymphocyte subsets were detected using the fluorescent monoclonal antibody reagent kit CD3-FITC/CD16+56-PE/CD45-PerCP-Cy5.5/CD4-PC7/CD19-APC/CD8-APC-Cy7 (the reagent kits were purchased from Tianjin Kuangbo Tongsheng Biotechnology Co., Ltd) via flow cytometry. Parameters assessed included: Percentage of total T lymphocytes (CD3^+^CD19^−^%), Percentage of total B lymphocytes (CD3^−^CD19^+^%), Percentage of helper/inducer T cells (CD3^+^CD4^+^%), Percentage of suppressor/cytotoxic T cells (CD3^+^CD8^+^%), Helper to suppressor (CD4^+^/CD8^+^) T cell ratio, and percentage of natural killer cells (CD3^−^/16^+^56^+^%), as well as the Absolute counts of: Total T lymphocytes (CD3^+^CD19^−^) cells/μl, Total B lymphocytes (CD3^−^CD19^+^) cells/μl, Helper/inducer T cells (CD3^+^CD4^+^) cells/μl, Suppressor/cytotoxic T cells (CD3^+^CD8^+^) cells/μl, and Natural killer cells (CD3^−^/16^+^56^+^) cells/μl.

#### Assessment of cytokines

On the examination day, a 3.5 mL blood sample was drawn from the participants’ antecubital vein into a gel-containing tube. Using a flow cytometry-based fluorescent immunoassay from the cytokine panel assay kit (the reagent kits were purchased from Tianjin Kuangbo Tongsheng Biotechnology Co., Ltd.), soluble proteins in the test sample were identified by recognizing microspheres of specific size and fluorescence intensity. The assay quantified levels of the following cytokines: IL-1β, IL-2, IL-4, IL-5, IL-6, IL-8, IL-10, IL-12p70, IL-17A, TNF-α, and IFN-γ.

#### Dizziness handicap inventory (DHI) scale

The DHI scale is widely used as symptomatic assessment tool in vestibular clinical practice (19). The impact of subjective symptoms of dizziness and balance disorders on daily life is evaluated. The original version of the DHI, consisting of 25 questions, was used to calculate four indices: the DHI total index and three DHI Subscales (DHI- Physical, DHI- Functional, DHI- Emotional). The DHI- Physical (DHI-P) assesses somatic factors, the DHI- Emotional (DHI-E) evaluates emotional factors, often associated with depression, and the DHI- Functional (DHI-F) assesses functional factors, primarily related to anxiety. The DHI total index ranges from 0 to 100, with higher scores indicating greater severity of subjective dizziness symptoms. The severity levels are categorized as follows: 0–30 (mild), 31–60 (moderate), and >60 (severe). The DHI-E Subscale ranges from 0 to 36, with the questions mostly reflecting depressive symptoms. The DHI-F Subscale ranges from 0 to 36 and is primarily associated with anxiety-related factors. The DHI-P Subscale ranges from 0 to 28 and primarily indicates physical elements.

All patients completed blood tests and initial DHI assessments within 1–3 days of disease onset, and the second DHI assessment was performed at discharge on days 7–10 post-onset.

#### Treatment regimen

During their hospital stay, Betahistine mesylate was administered orally at a dosage of 12 mg, three times a day (TID). Betahistine mesilate is primarily used to treat vertigo by improving blood flow in the inner ear, thereby reducing pressure and fluid buildup. It functions as a histamine H1 receptor agonist and H3 receptor antagonist. Gastrodin was given intravenously at a dosage of 600 mg once a day (QD). Gastrodin is derived from Gastrodia elata, a traditional Chinese medicinal herb, and is used for its neuroprotective and anti-vertigo effects. In addition, they underwent vestibular rehabilitation training. Importantly, no medications, including steroids, that might influence the outcomes of hematological assessments were given.

### Statistical analysis

Statistical analysis was performed using IBM SPSS 27.0. The median, mean and standard deviation represents quantitative parameter data, while frequency represents qualitative data. The Mann–Whitney *U* test was used to calculate the difference between two independent samples of nonparametric distributions, the Kruskal–Wallis *H* test was used to calculate the difference among several independent samples among nonparametric distributions. The Bonferroni correction method was applied for pairwise comparisons to adjust for multiple comparisons and reduce the risk of Type I errors. The chi-square test was used to compare qualitative data. The Spearman rank correlation coefficient test was used to analyze the correlation between different variables. A *p*-value less than 0.05 was considered statistically significant.

## Results

### Patient characteristics

**Figure 1:**
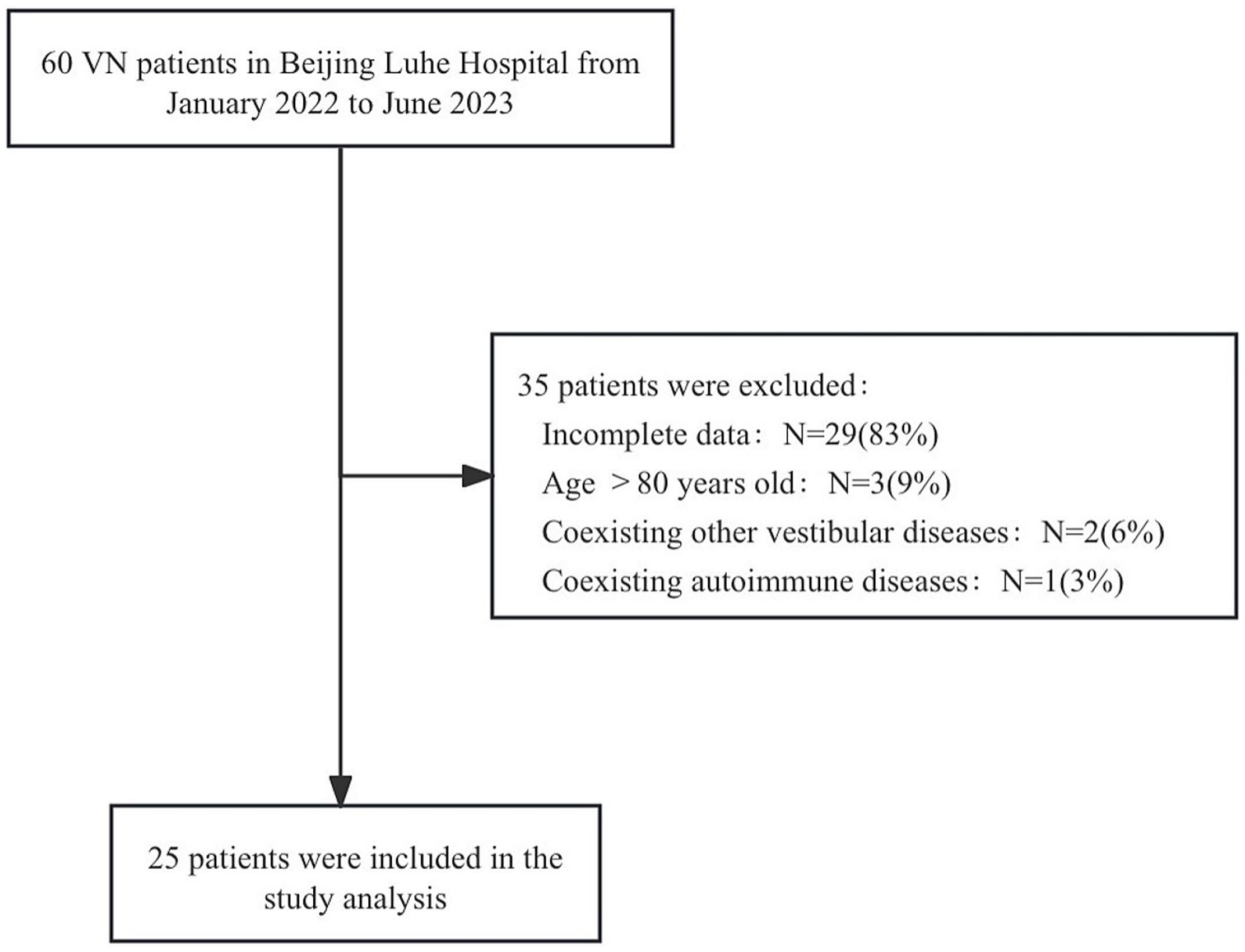
Study population.

In this study, we have identified 60 potential patients initially, out of which 35 were excluded due to incomplete data or failing to meet eligibility criteria, such as age limits, coexisting vestibular or autoimmune diseases. Thus, the final study sample included 25 eligible participants who met all criteria (Figure 1). This study involved 25 individuals diagnosed with VN and an additional 25 healthy participants forming the control group. The age of patient group ranged from 34 to 73 years (mean ± SD = 54.64 ± 11.99), and the age of control group ranged from 33 to 74 years (mean ± SD = 50.20 ± 10.89). In terms of gender distribution, the patient group had 13 males and 12 females, whereas the control group consisted of 7 males and 18 females. There was not a notable discrepancy in age or gender between the two groups (Table 1). Of the patients, 8% (or two individuals) reported a prior or concurrent viral infection, specifically in the upper respiratory tract. All patients were free from other neurological diseases. The only notable finding during the neurological evaluations was evidence of vestibular nerve dysfunction.

**Table 1:** Patient characteristics.

Group	Number	Male/Female	Age, mean (SD), years
VN	25	13/12	54.64 (11.99)
Control	25	7/18	50.20 (10.89)
*X*^2^/*U*		3.00	240.50
*p*		0.083	0.162

SD, standard deviation.

### Levels of peripheral blood lymphocyte subsets and cytokines in VN patients and healthy controls

The levels of peripheral blood total B lymphocytes (CD3^−^CD19^+^) (*p* = 0.017), and helper/inducer (CD3^+^CD4^+^) T cells (*p* = 0.006) were significantly higher in patients with VN than in the Healthy Controls (Table 2). The levels of peripheral blood total T lymphocytes (CD3^+^CD19^−^) (*p* = 0.002) and suppressor/cytotoxic (CD3^+^CD8^+^) T cells (*p* = 0.001) were significantly lower in patients with VN than in the Healthy Controls. The peripheral blood CD4^+^/CD8^+^ T cell ratio was significantly higher in patients with VN than in the Healthy Controls (*p* = 0.028). The levels of peripheral blood Natural Killer (NK) cells (CD3^−^/16^+^56^+^) (including percentage of NK cells *p* = 0.008 and absolute count of NK cells *p* = 0.000) were significantly lower in patients with VN than in the Healthy Controls (Table 3). The levels of IL-6 in the VN group were significantly higher than those in the Healthy Controls (*p* = 0.033), whereas the levels of IL-17A in the VN group were significantly lower than those in the Healthy controls (*p* = 0.029). These findings suggest that immune responses were involved in the pathogenesis of VN.

**Table 2:** Lymphocyte subsets in VN patients and healthy controls.

Lymphocyte subsets	CD3^−^CD19^+^%	CD3^+^CD4^+^%	CD4^+^/CD8^+^	CD3^+^CD8^+^	CD3^+^CD19^−^
VN	16.70 ± 5.38	42.93 ± 9.16	2.14 ± 0.97	406.68 ± 251.98	1234.28 ± 511.66
Controls	13.08 ± 5.92	35.66 ± 8.44	1.58 ± 0.68	648.32 ± 314.05	1757.92 ± 553.19
*U*	190.00	171.00	199.00	146.00	155.00
*p*	0.017	0.006	0.028	0.001	0.002

CD3^−^CD19^+^%: Percentage of total B lymphocytes. B lymphocytes are crucial for antibody production and humoral immune responses; CD3^+^CD4^+^%: Percentage of helper/inducer T cells. These cells are vital for evaluating cellular immune function; CD4^+^/CD8^+^: Helper/suppressor T cell ratio. This ratio assesses the balance and status of the immune system; CD3^+^CD8^+^: Absolute count of suppressor/cytotoxic T cells (cells/μl). These cells play a role in regulating immune responses; and CD3^+^CD19^−^: Total count of T lymphocytes (cells/μl). T lymphocytes are key players in immune regulation and response. U: the comparison of the sum of ranks between two sets of data; p: the probability of observing the sample data results.

**Table 3:** Lymphocyte subsets and cytokines in VN patients and healthy controls.

Lymphocyte subsets/cytokines	CD3^−^/16^+^56^+^%	CD3^−^/16^+^56^+^	IL-6	IL-17A	TNF-α
VN	8.56 ± 3.19	141.44 ± 59.18	5.23 ± 3.72	1.11 ± 2.25	1.82 ± 3.49
Control	13.30 ± 6.46	332.48 ± 170.20	3.19 ± 2.23	5.00 ± 7.67	1.01 ± 0.70
*U*	175.00	94.00	202.50	208.00	290.50
*p*	0.008	0.000	0.033	0.029	0.669

CD3^−^/16^+^56^+^%: Percentage of NK cells. NK cells are important for anti-tumor activity, viral defense, and immune regulation; CD3^−^/16^+^56^+^: Absolute count of NK cells (cells/μl). NK cells contribute significantly to immune system functions; IL-6: Level of Interleukin 6. IL-6 is key in inflammation and immune responses; IL-17A: Level of Interleukin-17A. IL-17A is important for immune system function and inflammation response; and TNF-α: Level of Tumor Necrosis Factor-alpha. TNF-α is involved in systemic inflammation and is a member of a group of cytokines that stimulate the acute phase reaction.

### CD3^+^CD4^+^ T cell levels and CD4^+^/CD8^+^ T cell ratio on the discharge DHI scores

**Figure 2:**
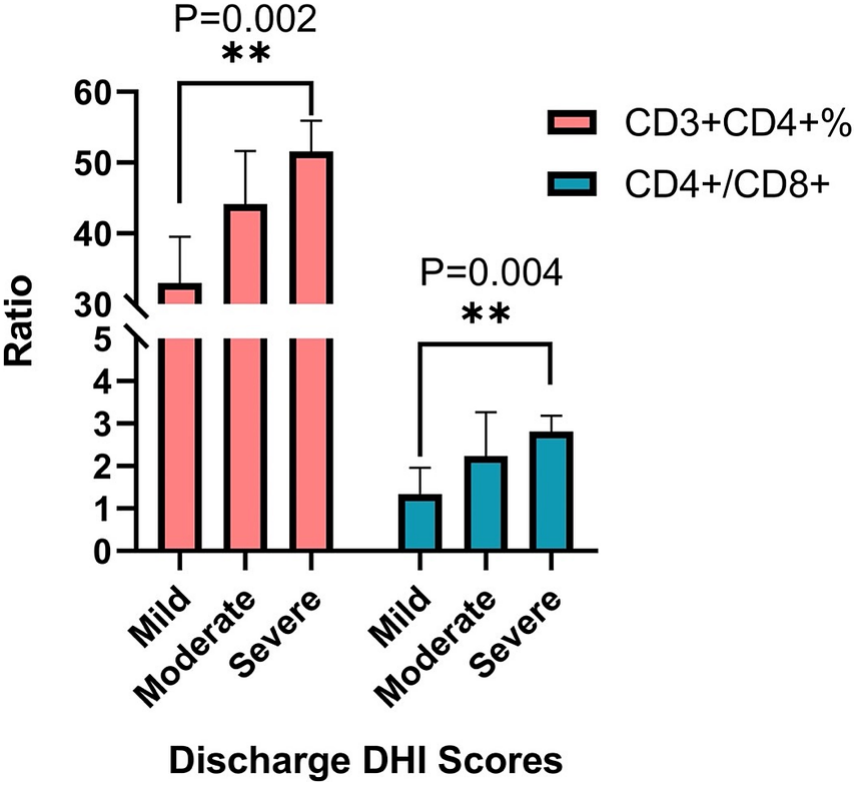
Pairwise comparison of CD3^+^CD4^+^ T cell levels and CD4^+^/CD8^+^ T cell ratio among different groups based on the discharge DHI scores. Adjusted significance values using the Bonferroni correction method.

The DHI total index ranges from 0 to 100, with higher scores indicating greater severity of subjective dizziness symptoms. The severity levels are categorized as follows: 0–30 (mild), 31–60 (moderate), and >60 (severe). All patients showed improvement in dizziness upon discharge compared to admission, including the total DHI score (80.5 ± 11.1 vs. 44.4 ± 20.4) (*p* = 0.00), DHI-P (27.4 ± 0.9 vs. 16.5 ± 5.5) (*p* = 0.00), DHI-F (34.7 ± 2.1 vs. 22.3 ± 8.3) (*p* = 0.00), and DHI-E (34.7 ± 2.1 vs. 5.6 ± 8.8) (*p* = 0.00). There are differences in the CD3^+^CD4^+^ T cell levels (*p* = 0.002) and CD4^+^/CD8^+^ T cell ratio (*p* = 0.006) among patients in the three groups of mild, moderate, and severe (Table 4), with the severe group having a significantly higher level and ratio than the mild group (*p* = 0.002 and *p* = 0.004) (Figure 2).

**Table 4:** CD3^+^CD4^+^ T cell levels and CD4^+^/CD8^+^ T cell ratio among different groups based on the discharge DHI scores.

Group	*N*	CD3^+^CD4^+^%			CD4^+^/CD8^+^		
		$\bar{X}\pm S$	*H*	*p*	$\bar{X}\pm S$	*H*	*p*
Mild	6	32.983 ± 6.550	11.984	0.002	1.340 ± 0.619	10.256	0.006
Moderate	14	44.111 ± 7.540			2.239 ± 1.023		
Severe	5	51.576 ± 4.344			2.818 ± 0.371		

### Levels of CD3^+^CD4^+^ T cells or CD4^+^/CD8^+^ T cell ratio and DHI scores

All patients completed the initial DHI assessment (include DHI-F1, DHI-E1, DHI-P1, DIH-Total1) within 1–3 days of onset; the second DHI assessment (include DHI-F2, DHI-E2, DHI-P2, DIH-Total2) was conducted at discharge 7–10 days post-onset. The DHI-P assesses somatic factors, the DHI-E evaluates emotional factors, often associated with depression, and the DHI-F assesses functional factors, primarily related to anxiety.

**Figure 3:**
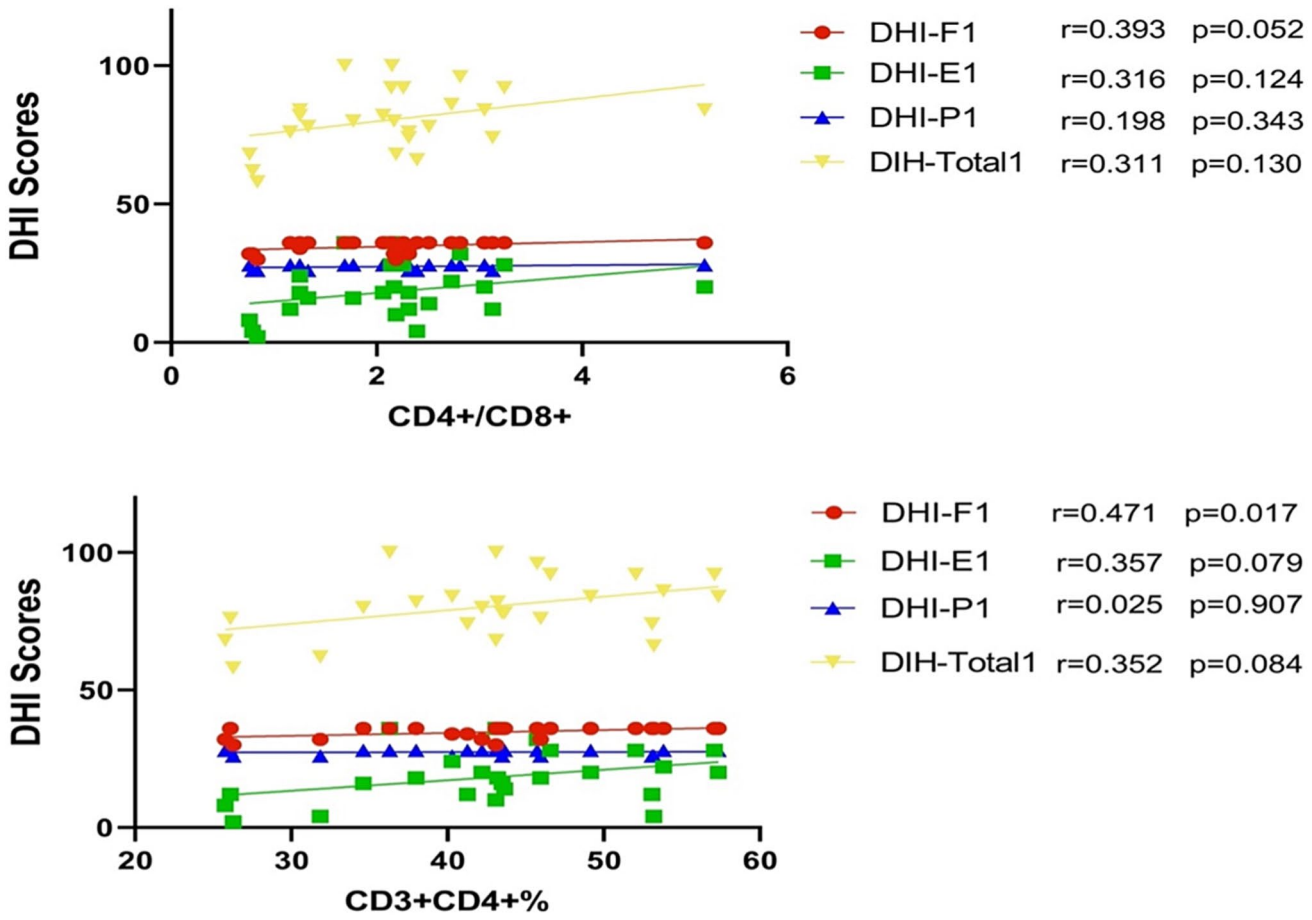
CD3^+^CD4^+^ T cells and CD4^+^/CD8^+^ T cell ratio correlated with DHI scores within 1–3 days of onset. The DHI subscales (DHI-Physical, DHI-Functional, DHI-Emotional) and the DHI Total Index (DHI-Total1) represent patient assessments within 1–3 days post-onset. Data show the relationship between immune cell subsets and the initial DHI scores.

**Figure 4:**
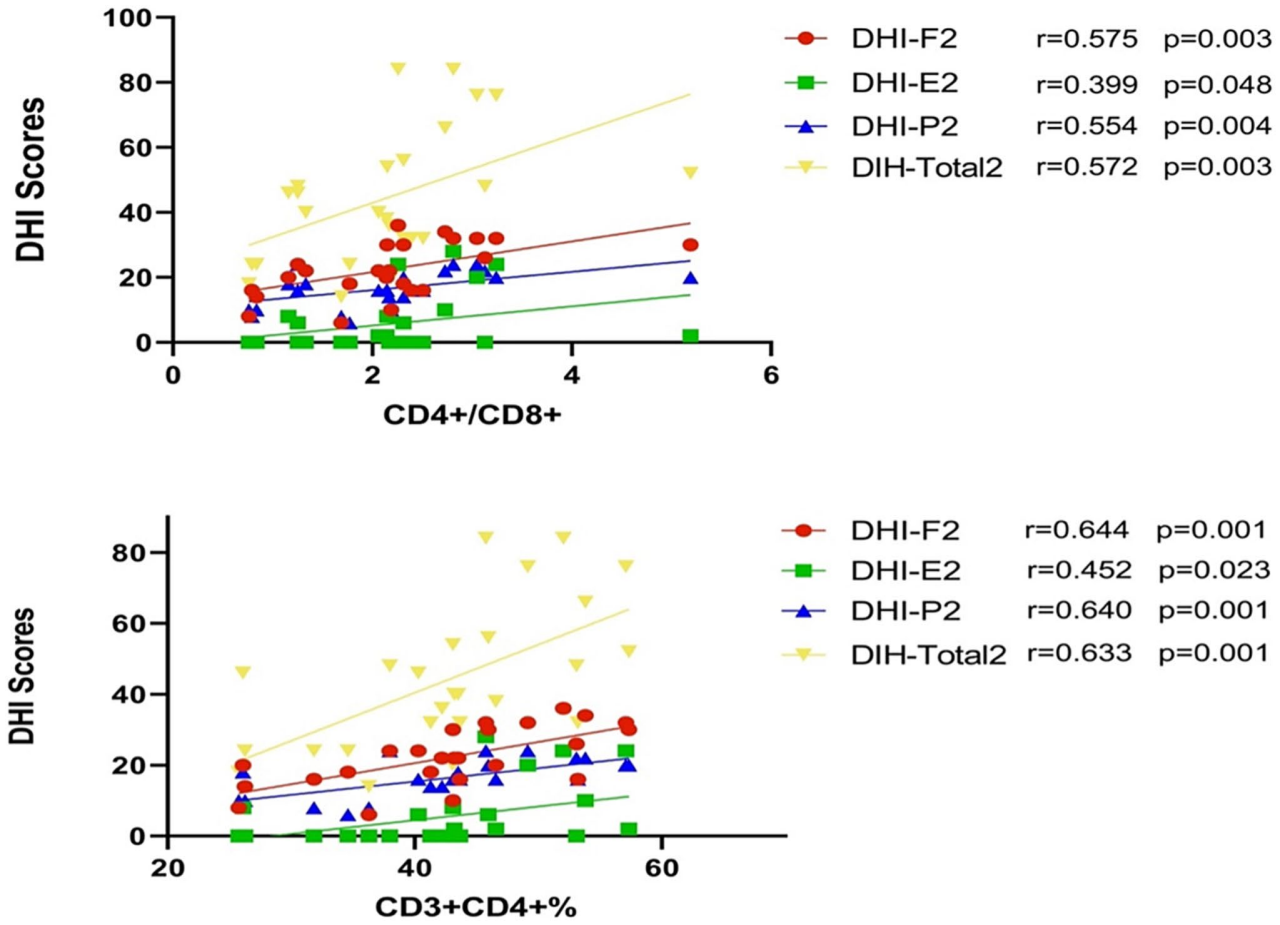
Correlation between levels of CD3^+^D4^+^ T cells, CD4^+^/CD8^+^ T cell ratio, and DHI scores at discharge (7–10 days post-onset). The DHI total index and three DHI subscales (DHI-Physical, DHI-Functional, DHI-Emotional) were assessed at discharge and represented as DHI-F2, DHI-E2, DHI-P2, and DHI-Total2. These data show the relationship between immune cell subsets and DHI scores at the discharge phase, 7–10 days post-onset.

The levels of CD3^+^CD4^+^ T cells on admission were positively correlated with the DHI-F (DHI- Functional) score within 1–3 days of onset (*p* = 0.017) (Figure 3). This result indicates an association of higher level of CD3^+^CD4^+^ on admission with higher DHI- Functional score and more severe anxiety and dizziness symptoms. Similarly, the levels of CD3^+^CD4^+^ T cells and the CD4^+^/CD8^+^ T cell ratio on admission were positively correlated with all DHI score (including DHI-F2, DHI-E2, DHI-P2, DHI-Total2) at discharge 7–10 days post-onset (all *p* < 0.05) (Figure 4). This indicates that higher levels of CD3^+^CD4^+^ T cells and CD4^+^/CD8^+^ T cell ratio on admission are associated with higher DHI scores at discharge, suggesting more severe dizziness symptoms and indicating a worse recovery.

## Discussion

In our study on VN, we employed flow cytometry to analyze peripheral blood T and B lymphocyte subsets, as well as cytokine levels. Our findings revealed significant differences in these immune parameters between VN patients and healthy controls. Specifically, VN patients showed increased levels of B lymphocytes, helper T cells, and a higher CD4^+^/CD8^+^ T cell ratio, decreased levels of T lymphocytes, suppressor T cells, and NK cells, along with elevated IL-6 and decreased IL-17A levels. Interestingly, the severity of VN symptoms correlated with specific immune markers. Patients with more severe VN had higher levels of CD3^+^CD4^+^ T cells and a higher CD4^+^/CD8^+^ T cell ratio. Additionally, higher levels of these markers at admission were linked to more severe symptoms and indicated a potential for slower recovery. In summary, the present study suggests that certain immune markers, particularly CD3^+^CD4^+^ T cells and the CD4^+^/CD8^+^ T cell ratio, could serve as indicators of VN severity and recovery prospects, offering insights into the disease’s immune-related pathogenesis.

Although the exact etiology of VN is unclear, viral infection, non-infectious inflammation or immune-mediated mechanisms are thought to be potential causes. Previous studies have been conducted to determine the pathogenesis of VN, but the results have been controversial. Initial theories linked VN to viral infection caused by the reactivation of neurotropic viruses such as herpes simplex virus type 1 (HSV-1) (4). Around 30% of affected patients had common colds before developing VN, according to research reports (5). In our study, we found only 2 (8%) patients with a history of viral infection, both of which were upper respiratory infections. Although vestibular neuritis (VN) has traditionally been attributed to viral reactivation, particularly herpes simplex virus type 1, accumulating recent evidence challenges this hypothesis. Antiviral therapy has not demonstrated clinical benefit (20), and a 2025 Mendelian randomization study found no causal association between multiple human herpesviruses and VN (21), arguing against a primary viral etiology. In recent years, increasing evidence suggests that non-infectious inflammation and immune response plays a significant role in VN pathogenesis. In 2011, the study found a significant elevation of percentage of proinflammatory CD40+, TNF-α+, COX-2+, CD38 + PBMCs in VN patients using Enzyme-Linked Immunosorbent Assay (ELISA), Ficoll density gradient isolation, and 2-color fluorescence – activated cell sorter analyses (6). Previous evidence (9) suggests that the neutrophil-to-lymphocyte ratio, percentage of CD40 + monocytes and macrophages were significantly higher in VN patients as compared to age-matched controls using gene expression profiling combined with bioinformatics analysis. This suggests that a neutrophil-mediated immune mechanism may play a critical role in the development of VN potentially promoting the disease and leading to inflammatory and thrombotic changes in the vestibular organ. Elevated neutrophil-to-lymphocyte ratios (NLR) (7–22) and platelet-to-lymphocyte ratios (PLR) (22) also suggest a possible non-infectious inflammatory etiology in VN. It has previously been reported that pathological CD4^+^/CD8^+^ T cell ratios appeared in 48% of VN cases using an immunofluorescence method, and the observed immunological imbalance is similar to the findings in multiple sclerosis. These findings support the hypothesis that VN is mediated by an immune response (8). Growing contemporary data also support an immune-mediated mechanism. Recent studies have identified immune abnormalities in acute unilateral peripheral vestibulopathy, including the presence of anti-ganglioside antibodies in a subset of patients (23). Moreover, increasing reports of VN following COVID-19 infection (5, 24, 25) or vaccination (26–30) suggest that immune dysregulation, rather than direct viral neurotropism, may act as a triggering factor. Within this evolving framework, our findings of altered T- and B-lymphocyte subsets and cytokine profiles, together with their association with symptom severity and short-term recovery, provide timely evidence supporting immune imbalance as a central mechanism in VN pathogenesis.

B lymphocytes are important immune cells that can secrete antibodies, present antigens, produce cytokines, and participate in important pathological and physiological processes such as immune regulation and inflammatory responses. B lymphocytes are associated with humoral immunity. T lymphocytes are an important component of the immune system. T lymphocytes control cellular immunity and possess antiviral and immune system regulatory functions. Their cell function depends on the relative composition of all T lymphocytes (CD3) and their subgroups (CD4, CD8). CD3^+^CD4^+^ T cells are helper/inducer cells. CD3^+^CD8^+^ T cells are suppressor/ cytotoxic cells (31). In our study, as compared to healthy subjects, patients with VN exhibited higher levels of total B lymphocytes (CD3^−^CD19^+^), CD3^+^CD4^+^ T cells and lower levels of total T lymphocytes (CD3^+^CD19^−^), CD3^+^CD8^+^T cells. These results suggest that immune responses may be involved in the pathogenesis of VN. Both B lymphocytes and T lymphocytes play a role in the pathogenesis of VN. Patients with VN exhibit immune dysfunction, although the detailed molecular mechanisms remain unclear.

The CD4^+^/CD8^+^ T cell ratio is a common marker typically used to detect whether the immunological function is normal or abnormal. Under normal circumstances, the subgroups antagonize each other and reach a balance; when the immune system is unbalanced, the cell numbers and ratios are altered, which leads to diseases. In our study, patients with VN showed an elevated CD4^+^/CD8^+^ T cell ratio. This finding is similar to the previous discovery (10), it differs from the alterations in T and B lymphocyte subsets observed in direct viral infections (increased CD8^+^ cells and decreased CD4^+^/CD8^+^ ratio) (8, 10–35). This finding supports the role of immune response in the pathogenesis of VN.

Previous evidence suggests a significant increase in the percentage of B lymphocytes and a significant decrease in the percentage of T lymphocytes in patients with Bell’s palsy (36). In other demyelinating diseases such as acute exacerbation of multiple sclerosis and acute phase of Guillain-Barré syndrome, similar peripheral blood lymphocyte subset changes have been observed (37, 38). Therefore, it is believed that these immunological changes may play an important role in the pathogenesis of the aforementioned diseases, rather than being secondary to neural tissue damage (37). The immunological similarities between Bell’s palsy and Guillain-Barré syndrome suggest that these two diseases share similar etiology and pathogenic mechanisms (36, 37). In our study, we also observed similar peripheral blood lymphocyte subset changes, specifically a decrease in T lymphocytes and an increase in B lymphocytes. Additionally, there is anatomical consistency between the vestibular nerve and the facial nerve. Based on these findings, it is hypothesized that the involvement of the autoimmune response may play a role in the pathogenesis of VN. It is speculated that VN may potentially be an autoimmune neurologic disorder similar to Bell’s palsy and Guillain-Barré syndrome. Immunological perturbations are implicated in pathogenesis of VN.

Natural Killer (NK) cells, are a type of lymphocyte that stands alongside T lymphocytes and B lymphocytes. As natural immune cells, NK cells participate in and maintain the stability of the body’s immune environment and normal immune function. When the quantity and function of NK cells become abnormal, it may further disrupt the immune function in body, leading to a series of pathological and physiological changes (39). In our study, we found that patients with VN exhibited a decreased level of NK cells compared to the healthy control group. This suggests the involvement of NK cells in the pathogenesis of VN, with a downregulation of NK cells expression observed in VN patients. Similar decreases in NK cell levels are also observed in other autoimmune diseases, such as Systemic sclerosis (SSc), Systemic lupus erythematosus (SLE), and Neuromyelitis optica spectrum disorder (NMOSD) (39–41). NK cell immunotherapy has been used for many diseases, including cancer and autoimmune diseases (41). This finding may provide a new perspective for the treatment of VN.

Interleukin-6 (IL-6) is a cytokine produced by various cell types. It can promote the proliferation and differentiation of B cells and T cells, as well as induce the activation and secretion of macrophages and granulocytes. Elevated levels of IL-6 have been associated with the occurrence of autoimmune diseases (14). Detecting and monitoring IL-6 levels can provide valuable information for clinical diagnosis and treatment. It can also help evaluate the immune status and degree of inflammation in the body. Interleukin-17A (IL-17A) is a protein produced by T cells and other immune cells. It is involved in various inflammatory and autoimmune diseases. IL-17A helps recruit and activate immune cells such as neutrophils and promotes the production of other pro-inflammatory cytokines (8, 10, 15–42). IL-17A is closely associated with autoimmune diseases such as psoriasis, rheumatoid arthritis, and inflammatory bowel disease, as well as chronic inflammation and tissue damage (16). In addition, IL-17A also plays a role in host defense against certain infections, especially in combating fungal and certain bacterial infections. IL-17A stimulates the production of antimicrobial peptides and helps recruit immune cells to the site of infection (43, 44). Taken together, IL-17A is a key participant in immune responses, playing a role in maintaining normal immune function as well as contributing to inflammation and autoimmune diseases. We found that the IL-6 levels in the VN group were higher than those in the healthy control group, while the IL-17A levels in the VN group were lower than those in the healthy control group. The elevated levels of IL-6 and decreased levels of IL-17A may represent the activities of the operative factors of the cell-mediated immune system. It is speculated that IL-6 and IL-17A may play a role in VN, although their exact molecular mechanisms still require further research. In the present study, we did not find significant differences in other cytokines, such as IL-1β, IL-2, IL-4, IL-5, IL-8, IL-10, IL-12p70, TNF-α, and IFN-γ, suggesting that these cytokines may not play a key role in the acute phase of VN. Further studies are warranted to clarify this assumption.

The course of VN varies among patients, with most experiencing a marked improvement in severe vertigo within the first few days, followed by a gradual resolution of residual symptoms over subsequent weeks (1). However, a notable proportion of VN patients face the risk of developing chronic conditions. It has previously been shown approximately 15–30% of VN patients continue to experience persistent dizziness and visual oscillations even 1 year after the initial onset (45). Similarly, previous evidence observed that over 30% of patients suffer from prolonged dizziness lasting more than a year.

The persistence of chronic dizziness in VN patients can be attributed to a multitude of factors (1). These include inadequate central compensation, where the brain’s ability to adapt to the imbalance caused by VN is insufficient, and incomplete peripheral recovery, referring to the ongoing dysfunction in the vestibular system. Additionally, psychophysiological and psychological factors, such as anxiety or stress responses triggered by the initial VN episode, can exacerbate or prolong the experience of dizziness. Understanding these diverse contributing factors is crucial for effectively managing and treating chronic symptoms in VN patients.

While vestibular function tests were used for the diagnosis of VN and assessment of vestibular function (46, 47), these tests may not predict the clinical recovery of patients (48–50). Our study found that the levels of the CD3^+^CD4^+^ T cells and CD4^+^/CD8^+^ T cell ratio in patients with VN differed among different patient groups, with significantly higher levels in the severe group than in the mild group. The novelty of this study lies in its integrated immune profiling of VN patients, combining T and B lymphocyte subsets with cytokines, and correlating these with clinically relevant DHI scores. To our knowledge, this is the first report to demonstrate that CD3^+^CD4^+^ T cell levels and CD4^+^/CD8^+^ T cell ratio are not only altered in VN but also correlate with symptom severity and short-term prognosis. These findings provide new evidence supporting an immune-mediated mechanism in VN and suggest potential biomarkers for clinical prognosis.

### Perspective and prospective

In conclusion, immunological perturbations are implicated in pathogenesis of VN. Evaluation of these immune markers could offer insights into prognostic outcomes for VN patients, leading to development of therapeutic strategies. It is worth considering whether immunomodulatory treatment can be used to facilitate faster recovery of clinical symptoms in the clinical management of VN. It seems logical that corticosteroids which exert their beneficial effects through immunosuppressive actions, may be beneficial in this regard (51).

This investigation is a retrospective study with a relatively small sample size, which may not fully represent the larger population. We did not monitor changes in immune cell cytokines at different stages of VN, which could provide more comprehensive insights. The use of the DHI for VN assessment, while common, may not fully capture the acute and fluctuating nature of the condition, and future studies should explore alternative or complementary measures. Future studies will require a prospective design, incorporating larger, multicenter cohorts to validate these findings. Additionally, we will aim to explore the relationship between immune cell changes and long-term prognosis, as well as track cytokine variations throughout the different stages of VN, including the alterations in immune cell cytokine levels in patients with VN following immunotherapy, such as glucocorticoids.

## Data Availability

The raw data supporting the conclusions of this article will be made available by the authors, without undue reservation.
